# PharmaKoVariome database for supporting genetic testing

**DOI:** 10.1093/database/baac092

**Published:** 2022-10-18

**Authors:** Jungeun Kim, Jae-Pil Choi, Min Sun Kim, Jong Bhak

**Affiliations:** Personal Genomics Institute (PGI), Genome Research Foundation (GRF), Cheongju 28190, Republic of Korea; Personal Genomics Institute (PGI), Genome Research Foundation (GRF), Cheongju 28190, Republic of Korea; Personal Genomics Institute (PGI), Genome Research Foundation (GRF), Cheongju 28190, Republic of Korea; Personal Genomics Institute (PGI), Genome Research Foundation (GRF), Cheongju 28190, Republic of Korea; Korean Genomics Center (KOGIC), Ulsan National Institute of Science and Technology (UNIST), Ulsan 44919, Republic of Korea; Department of Biomedical Engineering, School of Life Sciences, UNIST, Ulsan 44919, Republic of Korea; Clinomics, Inc., Ulsan 44919, Republic of Korea

## Abstract

Pharmacogenomics (PGx) provides information about routine precision medicine, based on the patient’s genotype. However, many of the available information about human allele frequencies, and about clinical drug–gene interactions, is based on American and European populations. PharmaKoVariome database was constructed to support genetic testing for safe prescription and drug development. It consolidated and stored 2507 diseases, 11 459 drugs and 61 627 drug–target or druggable genes from public databases. PharmaKoVariome precomputed ethnic-specific abundant variants for approximately 120 M single-nucleotide variants of drug–target or druggable genes. A user can search by gene symbol, drug name, disease and reference SNP ID number (rsID) to statistically analyse the frequency of ethnical variations, such as odds ratio and *P*-values for related genes. In an example study, we observed five Korean-enriched variants in the *CYP2B6* and *CYP2D6* genes, one of which (rs1065852) is known to be incapable of metabolizing drug. It is also shown that 4–6% of North and East Asians have risk factors for drugs metabolized by the *CYP2D6* gene. Therefore, PharmaKoVariome is a useful database for pharmaceutical or diagnostic companies for developing diagnostic technologies that can be applied in the Asian PGx industry.

**Database URL**: http://www.pharmakovariome.com/

## Introduction

Pharmacogenomics (PGx) provides routine precision medicine by considering a patient’s genetic background (1). The declining costs of next-generation sequencing technologies are accelerated by the development of genomic profiles, such as single-nucleotide variants (SNVs) and copy number variants, from a large population. Population variome resources allow constructing genotyping panels to analyse drug metabolism or gene–drug pairing functionality. The Clinical Pharmacogenetics Implementation Consortium (CPIC) provides terminology standards for clinical pharmacogenetic test results (2). This database provides clinical evidence of the pharmacogene and corresponding drug interactions (3). PharmVar has initially analysed cytochrome P450 (CYP) enzymes that function as drug metabolizers and later expanded to other pharmacogenes (4). PGx has also been involved in *in silico* approaches in several cancer genomics. Rich oncogenomics databases allow for genetic marker-based cancer therapy (5–7). EURO-PGx, a consortium of European research initiatives, has developed 1931 PGx biomarkers among the European population (8–10). Even today, variome data for diagnosis and treatment of disease cohorts are steadily accumulating.

Despite Asians (4.6 billion populations) comprising more than 60% of the global population, there is a deficiency of PGx data on Asian populations (11). Currently, the Southeast Asian (SEA) Pharmacogenomics Research Network was established in 2018 (12) and constructed a SEA panel of 100 PGx loci to interpret variations in drug response phenotypes (13). However, East Asian (EA) populations are genetically different, having originated from the admixture of different ancestral populations (14). This suggests that the EA population differs in genetic epidemiology and in the diversity of risk alleles for drugs. This necessitates the characterization of the EA-PGx variome. In addition, the PGx market for EA populations, currently numbering 1.7 billion, is still large. We constructed a Korean variome database, named ‘KoVariome’ (15) and updated it up to 1094 individuals (16). EA comprises three countries: Korean, Chinese and Japanese. Korea is located between China and Japan, and the populations of these countries are genetically similar because of their shared ancestors (14). Therefore, the KoVariome database is suitable to provide a PGx panel representative of the EA subpopulation by comparing the genetic difference and allele frequencies with global genetic variations (1000 genomes project, 1KGP) (17) and variations for other Asian subpopulations (18).

To successfully implement EA-PGx, the PharmaKoVariome database provides EA-specific variants and variant pathogenicity of pharmacogenes that are differentially distributed across subpopulations worldwide. This facilitates the screening of essential variants for PGx implementation in the EA population but is not limited to EA. PharmaKoVariome can be accessed by searching for each pharmacogene, drug and disease, and these three pieces of information are cross-linked in the database. Such information is useful for pharmaceutical researchers to simply and economically design assay developments to test allele-specific adverse drug reactions, select drugs and adjust doses. Therefore, PharmaKoVariome can be useful for constructing EA-PGx panels and developing precision medicines before clinical trials.

## Methods and data description

### Data resources

**Figure 1.**
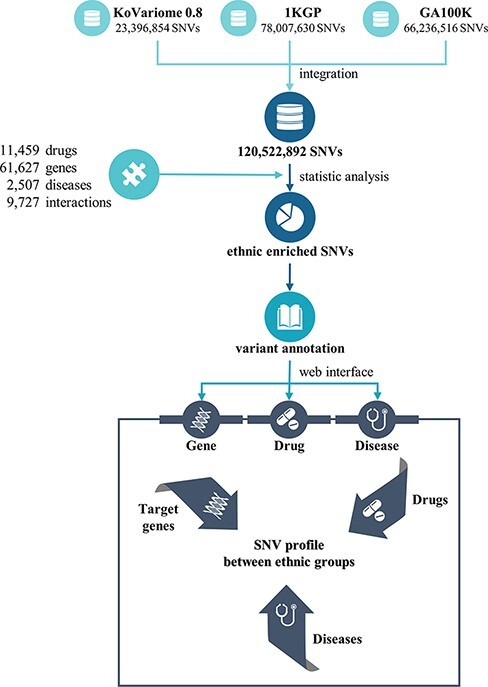
Flowchart to construct the PharmaKoVariome database.

PharmaKoVariome is a PGx database that provides SNV frequencies of drug–target or drug metabolic genes in 12 human ethnic groups worldwide to construct national guidelines for precision medicine. It integrated 121 M SNVs generated from three variome databases, such as KoVariome, 1KGP and Genome Asia 100K, including 12 ethnic groups (Table 1 and Figure 1). Ethnicity-enriched SNVs were precalculated based on Fisher’s exact test of SNV frequencies, which enables PGx clinical research to be focused on the national haplotype structures. PharmaKoVariome also provides putative clinical significance (pathogenicity) for each SNV, pre-estimated by PolyPhen-2 and sorting intolerant from tolerant (SIFT) results in dbNSFP (ver. 2.9.1) (19). To provide genetic variations in pharmacogenes, we integrated 61 627 drug–target or druggable genes based on the Entrez_id, which were collected from the drug–gene interaction database (ver. 4.0) (20) and therapeutic target database (ver. 7.1.01) (21). Based on the genomic loci [with reference to hg19 (22)] of these genes, PharmaKoVariome provides allelic variation and frequency and precalculated statistics of each SNV. We also collected information on 11 459 drugs and 2507 diseases and categorized them based on the unique code of the International Classification of Disease-11 generated from Kyoto Encyclopedia of Genes and Genomes (KEGGs) databases (23). To help with the design of clinical studies specific to nations, PharmaKoVariome has linked drug and disease information to pharmacogene, with comparable variation frequencies and statistics. These integrated pharmacological data can be used for clinical studies in Asia and other countries, which have been relatively neglected in drug development and clinical research.

**Table 1.** Statistics of PharmaKoVariome

	Statistics
No. of druggable genes	37 462
No. of SNV loci	1 428 986
Synonymous	533 032
Non-synonymous	867 063
Splice acceptor	7017
Splice donor	8816
Start lost	2164
Stop gained	18 717
Stop lost	1006

## Results and discussion

### Usage of PharmaKoVariome

**Figure 2.**
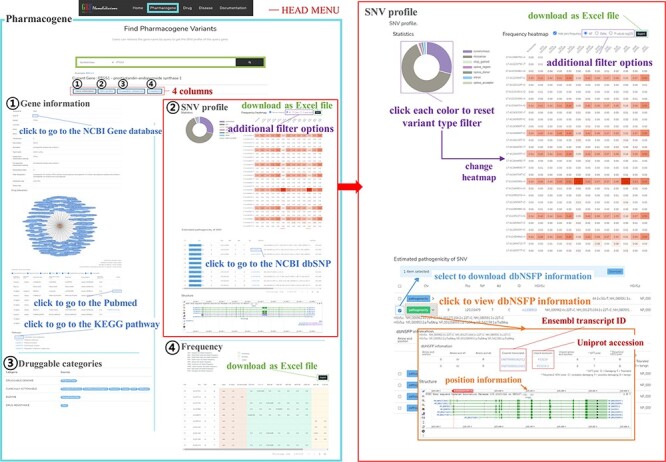
Example of the PharmaKoVariome usage.

PharmaKoVariome allows users to retrieve information by querying (i) gene, (ii) drug and (iii) disease name (Figure 2). Because the major goal of this database is to provide national variation statistics for pharmacogenes, the database was constructed to use ethnically different SNV frequencies for pharmacogenes, by searching with gene names, alias or reference SNP ID number (rsID) for pharmacogene (Figure 2a). PharmaKoVariome provides general information about the pharmacogene in the ① ‘gene information,’ including drug interaction and KEGG pathways. The ② ‘SNV profile’ provides precalculated statistics for each SNV locus of pharmacogene (see details below). The ③ ‘Druggable categories’ and ④ ‘Frequency’ provide gene categories and links for other databases and raw data of allele frequencies of SNV data, respectively. Users can access the ethnically different SNV frequencies for each locus of pharmacogene observed in ② ‘SNV profile’ tab (Figure 2b). PharmaKoVariome categorized SNVs into synonymous, missense, stop gained, stop lost, start lost, splice region, splice donor, splice acceptor and intron. These statistics are represented by pie chart, which can also be used to filter variants required for clinical studies. Pie chart sectors are clickable and provide information on the category (e.g. missense) that they represent. PharmaKoVariome shows heatmaps based on precalculated SNV abundance in each ethnic group. SNV abundance enables the user to select with three types of statistic values: (i) allele frequency, (ii) odds ratio and (iii) -log_2_(*P*-value). These statistics are useful to select the criterion for SNV filtering. User-filtered SNVs are exported as an Excel file. In addition, PharmaKoVariome provides pre-estimated pathogenicity and ClinVar information. By checking the select box for each variant, the user could download selected SNVs.

### Pharmacogenes with Korean-enriched pathogenic SNVs

**Figure 3.**
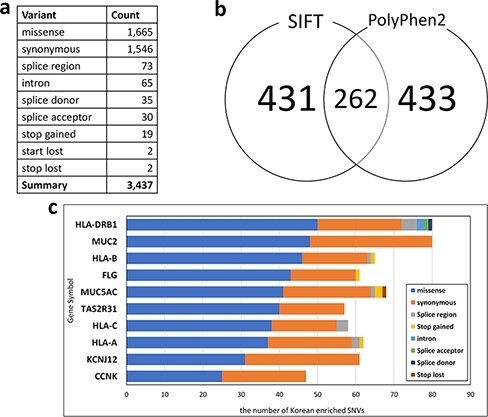
Statistics of Korean-enriched SNVs. (a) Annotation of Korean-enriched SNVs, (b) Venn diagram of the Korean-enriched pathogenic variants predicted by SIFT and PolyPhen-2 algorithms and (c) top 10 genes with Korean-enriched variants. The *x*-axis represents the number of Korean-enriched SNVs, and the *y*-axis represents the gene symbol.

For Korean PGx studies, we identified 3437 Korean-enriched SNVs from 1144 pharmacogenes with the criterion *P*-value < 0.001 and odds ratio > 3 (Figure 3a). The most abundant SNV type was missense mutation (1665 SNVs), which constituted 48.44% of Korean-enriched SNVs. In Korean-enriched SNVs, we also identified 19 stop-gained SNVs and 65 SNVs located in the splice donor or acceptor regions. These SNVs are possibly pathogenic by the destruction of canonical gene structures. We identified pathogenicity of 693 and 695 Korean-enriched missense SNVs by SIFT and PolyPhen-2 algorithms, respectively (Figure 3b). Among them, 262 Korean-enriched pathogenic SNVs were shared by SIFT and PolyPhen-2. A total of 178 PGx genes contained at least one Korean-enriched pathogenic SNV. The Human leukocyte antigen (HLA) gene family (HLA-DRB1, HLA-C and HLA-A) had a high number of Korean-enriched pathogenic missense SNVs (Figure 3c).

### Application of the PharmaKoVariome to identify Korean-enriched pathogenic variants in the *CYP* gene family

**Figure 4.**
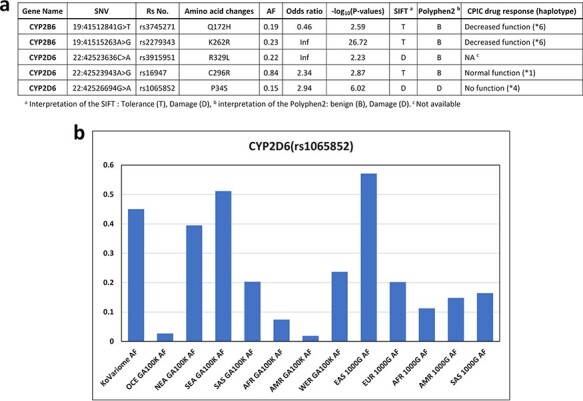
Korean-enriched missense SNVs in the *CYP* gene family. (a) Example of Korean-enriched missense variants in the *CYP* gene family and CPIC information. (b) Example of different allele frequencies for rs1065852 SNV in each ethnic group. The *x*-axis represents ethnicity and the database that contained the information, and the *y*-axis represents allele frequencies.

We examined Korean-enriched missense SNVs in the five *CYP* gene families, such as *CYP1A2*, *CYP2B6*, *CYP2C8*, *CYP2C9*, *CYP2C19*, *CYP2D6* and *CYP3A4*, which function in drug metabolism (Figure 4a). Among them, two SNVs were specific to the Korean population. The SIFT algorithm estimated that two SNVs (rs3915951 and rs1065852) are likely ‘dangerous,’ and PolyPhen-2 estimated that rs1065852 is pathogenic SNV (Figure 4a). We manually examined drug response of these SNV loci in CPIC database. Among five SNVs enriched in Korean population, four SNVs were examined for the effects on drug metabolism. One Korean-enriched missense SNV had no information about drug metabolism in CPIC database. Another SNV was functioning normally. However, two of these SNVs (rs374527 and rs227934) decreased drug metabolic function, and one SNV (rs1065852) cannot metabolize drugs. According to PharmaKoVariome database, at least 1–2% of Koreans have reduced or no ability to metabolize certain drugs. In addition, these alleles cause severe adverse effects depending on the function of the drug; therefore, PGx-based prescription is required. We examined allele frequency of rs1065852 SNVs (no function for drug metabolism) in other ethnic groups (Figure 4b). This showed frequency differences between the North Asian and EA population and other populations. Approximately 4–6% of North Asians and EA have genetic risk factors for drugs that are degraded by *CYP2D6* gene. Therefore, pharmaceutical or diagnostic companies must ensure that the information about the drugs metabolized by these genotypes is comprehensive and accurate and develop diagnostic technologies to utilize them in the Asian PGx industry.
